# Antimicrobial Susceptibility Testing of the Combination of Aztreonam and Avibactam in NDM-Producing *Enterobacterales*: A Comparative Evaluation Using the CLSI and EUCAST Methods

**DOI:** 10.3390/antibiotics14070675

**Published:** 2025-07-03

**Authors:** Linda Mei-Wah Chan, Doris Yui Ling Lok, River Chun Wai Wong, Alfred Lok-Hang Lee, Ingrid Yu-Ying Cheung, Christopher Koon-Chi Lai, Viola C. Y. Chow

**Affiliations:** 1 Department of Microbiology, Prince of Wales Hospital, Sha Tin, New Territories, Hong Kong SAR, Chinallh849@ha.org.hk (A.L.-H.L.);; 2 Department of Microbiology, Faculty of Medicine, The Chinese University of Hong Kong, Hong Kong SAR, China

**Keywords:** aztreonam-avibactam, broth-disk elution test, BDE, carbapenem-resistant *Enterobacterales*, carbapenemase-producer, CRE, CPE, metallo-beta-lactamase, MBL, NDM

## Abstract

**Background**: The combination of aztreonam (ATM) and avibactam (AVI) presents an important therapeutic option for carbapenem-resistant *Enterobacterales*, particularly the NDM-producing *Enterobacterales*. In 2024, both the CLSI and EUCAST published their methods in antimicrobial susceptibility testing for this combination of agents. **Materials and Methods**: Forty carbapenem-resistant *Enterobacterales* isolates, including *Escherichia coli* (*n* = 35), *Enterobacter cloacae* complex (*n* = 2), *Klebsiella pneumoniae* complex (*n* = 2), and *Citrobacter freundii* complex (*n* = 1) were included in this study. All isolates harbored the NDM carbapenemase except one, which had no known detected carbapenemases. Four antimicrobial susceptibility testing methods of the combination of ATM and AVI were evaluated on these isolates, including the CLSI broth disk elution (BDE) method, the disk diffusion (DD) method of aztreonam–avibactam (AZA) following the EUCAST breakpoints, the MIC test strip (MTS) method of AZA following the EUCAST breakpoints, and the gradient strip stacking (SS) method. BDE was used as the standard of comparison. **Results**: Using BDE as the standard of comparison, the AZA DD, AZA MTS, and SS methods had 100% categorical agreement (CA), 0% very major error (VME), and 0% major error (ME). The essential agreement (EA) between the AZA MTS and SS method was 57.5%. **Conclusions**: The AZA DD, AZA MTS, and the SS methods showed complete concordance with the BDE method. However, the MICs obtained from the AZA MTS and SS were not comparable.

## 1. Introduction

Antimicrobial resistance (AMR) is a major global public health threat, having contributed to 4.95 million deaths in 2019. The 2024 World Health Organization (WHO) list of bacterial priority pathogens have urged the prioritization of critical group carbapenem-resistant *Enterobacterales* (CRE) [1]. In the United States alone, CRE clinical isolates have seen a drastic increase in metallo-β-lactamase (MBL) genes from 4% to 20% between 2019 and 2021 [2]. Amongst these, the emergence of the New Delhi metallo-β-lactamase (NDM) has quickly become one of the most prevalent carbapenemase genes with global distribution [3,4,5,6,7,8].

The preferred treatment of MBL-producing *Enterobacterales* including NDM-producing *Enterobacterales* (NDM-E), is the combination of aztreonam (ATM) and avibactam (AVI) [4,5,6,7,8,9,10]. Various methods have been described for the AST of this drug combination. The gold standard broth microdilution method (BMD) is not easily accessible for routine microbiological use and is usually reserved for reference laboratory use. Other existing methods, including disk diffusion (DD), the MIC test strip (MTS), gradient strip stacking (SS), and combination MTS/DD methods, have not been standardized across laboratories [11,12,13]. In 2024, both the CLSI and EUCAST have updated new methods for determining the susceptibility of *Enterobacterales* to the combination of ATM and AVI. The CLSI adopted the broth disk elution (BDE) method previously described by Harris et al. [14,15]. The EUCAST has also published MIC and DD breakpoints for aztreonam–avibactam (AZA) [16].

In this study, we evaluate antibiotic susceptibility testing (AST) for the combination of ATM and AVI. Using methods accessible to clinical laboratories, BDE is used as the standard of comparison to compare DD, MTS, and SS.

## 2. Results

### 2.1. Comparison with Broth Disk Elution Test

Thirty-nine isolates were susceptible to the combination of CZA and ATM using the BDE test. One isolate was resistant to the combination of CZA and ATM in the BDE test.

All three methods, DD, MTS, and SS, showed identical susceptibility interpretation results with the BDE test, yielding a CA of 100%, VME of 0%, and ME of 0% (Table 1). The concordance rate of BDE reading between the two scientists was 100%.

### 2.2. Comparison of MIC Distribution for AZA Disk Diffusion Test, AZA MIC Test Strip, and Gradient MIC Test Strips

**Figure 1:**
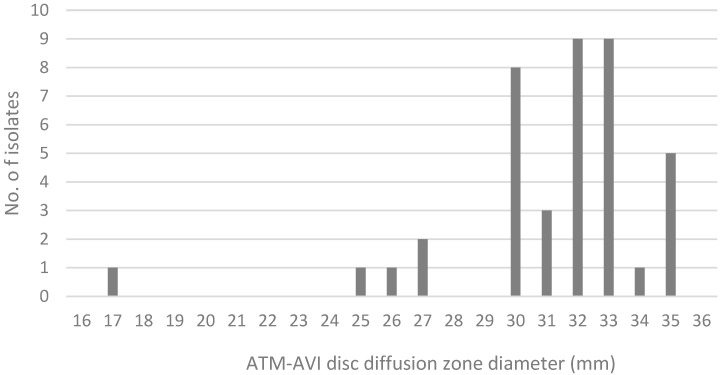
Distribution of Aztreonam–avibactam disk diffusion zone diameter.

Based on the AZA DD test, all isolates are found to be susceptible with zone diameters of 25 mm and above; no isolates fell within the area of technical uncertainty (ATU) (22 to 24 mm). One resistant isolate had an inhibition zone of 17 mm. The distribution of the AZA DD zone sizes is illustrated in Figure 1.

While the CA between the AZA MTS and SS was also 100%, there was significant variability between the MICs obtained from the two methods. The EA between the two methods was 57.5% (23/40). The comparison of MICs between the AZA MTS and SS is shown in Table 2.

**Figure 2:**
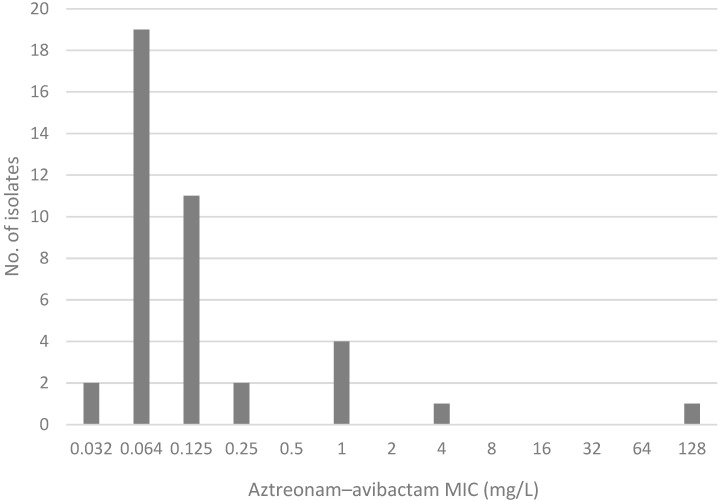
Aztreonam–avibactam MIC distribution by MIC test strip.

**Figure 3:**
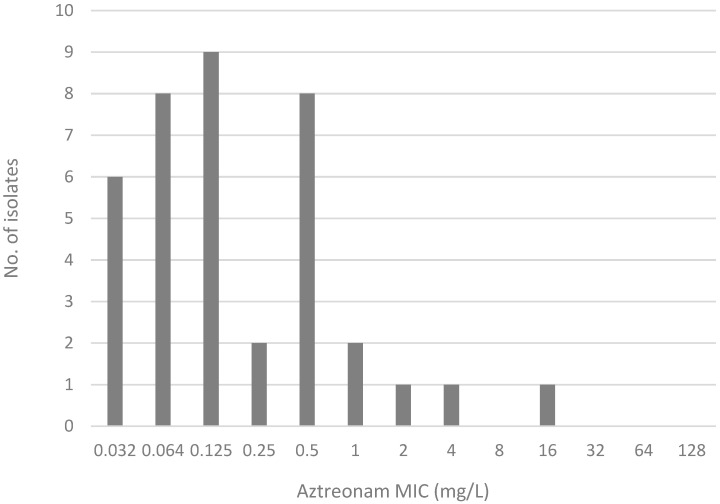
Aztreonam MIC distribution in the gradient strip stacking test.

The MIC distribution of AZA by the MTS, and of ATM by the SS test is shown in Figure 2 and Figure 3, respectively.

## 3. Discussion

This is the first study to compare the CLSI and EUCAST AST methods for testing the combination of ATM and AVI, as far as the authors are aware. The advent of the CLSI BDE method enables it to serve as a standard of comparison for evaluating non-standardized testing methods when the BMD reference method is not feasible. In this evaluation, we have found that the three methods of DD, the MTS, and SS have 100% CA for NDM-E.

### 3.1. Clinical Isolates

The clinical isolates are representative of the CRE strains in our local epidemiology, commonly comprising NDM-E. A variety of *Enterobacterales* were included to show that the methods evaluated were valid for different species in this order, with the majority being *Escherichia coli* isolates.

### 3.2. Comparison of Methods for Routine Laboratory Use

The methods used in this study are accessible to non-reference clinical laboratories. We used the CLSI BDE method as a standard of comparison to compare DD and the MIC test strips. Our finding of 100% CA for all methods supports the use of other laboratory methods with consideration of individual test characteristics, aside from BDE and BME, which are complex to perform routinely.

The methods used in this study including BDE, DD, and the MIC test strips, all require an incubation time of 16 to 20 h before result reading. BDE requires longer prior hands-on time, including a 30 min disk elution step. The SS method involves manual precision to remove and replace the MIC strips from the agar within 5 min. In comparison, the direct application of the disks and MIC strips to agar plates is faster with technical ease. BDE also requires more bench space and materials. For the testing of one isolate, one needs 20 CAMHB tubes (1 test strain and 4 control strains; 4 tubes for each strain). One opportunity for simplification would be to use one tube for testing CZA–ATM synergism, combined with another method, such as the disk diffusion test, for individual CZA and ATM testing instead.

The accuracy of the results can be limited by subjective interpretation. This is crucial for BDE turbidity reading, where accurate reading of all four tubes is necessary for confident results interpretation. Our scientists were able to reach a unanimous agreement for all isolates tested, nonetheless.

The MIC test strips are also known to have interpretation challenges as well, which is a possible explanation for the low EA rates for the AZA MIC in MTS and ATM MIC in SS. Current studies using BMD as a reference method have reported that for *Enterobacterales,* the MIC values obtained with the SS method tend to be higher to those obtained with the MTS method [17]. This is also observed in our study to a lesser extent. Several other studies generally demonstrate an overall better performance of the MTS method over SS [17,18,19,20]. This could be due to the additional technical precision required in SS.

### 3.3. Clinical Application for Guiding Antibiotic Use

In vitro susceptibility testing informs the clinical choice of antibiotics. The dosage of antibiotics related to the clinical breakpoints between the CLSI and EUCAST is different. The CLSI’s susceptibility testing is based on the dosing of 1 g of aztreonam intravenously every 8 h and 2.5 g of ceftazidime–avibactam (2 g CAZ, 0.5 g AVI) over 2 h intravenously every 8 h [14]. The EUCAST’s susceptibility is based on the dosing of 2 g aztreonam–avibactam (ATM 1.5 g, AVI 0.5 g) over 3 h intravenously every 6 h. The most commonly prescribed CZA–ATM dosing regimen is 2.5 g/2 g given intravenously every 8 h. The IDSA guideline recommends 2.5 g of CZA every 8 h and 2 g of ATM every 6 h [10]. In both scenarios, the dosage of the ATM component is higher than that of the CLSI. This would have implications for the interpretation and application of these clinical breakpoints.

The testing for the combination of ATM and AVI could be expanded to other multi-drug-resistant non-fermenters, including *Pseudomonas aeruginosa* and *Stenotrophomonas maltophilia.* The CLSI BDE method encompasses susceptible breakpoints for *Enterobacterales* (≤4/4 μg/mL), *Pseudomonas aeruginosa* (≤8/4 μg/mL), and *Stenotrophomonas maltophilia* (≤8/4 μg/mL) [10]. Additional challenges include the lack of DD breakpoints from both the EUCAST and CLSI, and lower reproducibility for *Pseudomonas aeruginosa* [17].

### 3.4. Limitations and Future Directions

There are some limitations in this study. Firstly, our study findings are based on a small sample size of forty isolates. A larger sample size could provide more robust statistical analysis to reinforce the positive findings of 100% CA across methods, in addition to exploring the low EA rates between the MIC test strip methods.

Secondly, most of the isolates were NDM-E. While this may represent a restriction in the potential spectrum of MBL producers, we reason that NDM-E that are *E. coli* comprise the largest group of MBL-E in our locality [5,7,8], representing the real-world scenario we face in daily laboratory practice and the wider region. ATM–AVI resistance is described predominantly amongst *E. coli* [17,19,20,21]. Our study aligns with this, demonstrating that for NDM-*E. coli*, DD or the MIC test strips are equally viable options. However, there should be caution in generalizing applications as there can be strain-dependent performance variabilities [17,18,19,20,22,23].

Thirdly, certain tests were sourced from different manufacturers due to availability reasons and are of routine laboratory use. This includes the MIC test strips for CZA and ATM from bioMérieux, diffusion disks for CZA and ATM from Oxoid, and both AZA MIC strips and diffusion disks from Liofilchem. The antibiotic disk concentrations and methods are according to the CLSI (CZA and ATM), and EUCAST (AZA) guidelines. Different manufacturers for CZA and ATM disks have been shown to affect the BDE results in the original study [15], which may be related to differences in elution physics amongst disks despite the same antibiotic concentration in disks. The manufacturers sourced in this study differ from those of concern in the original study, and the significance of sourcing from different manufacturers is uncertain in this study. Until this is clarified in future studies, further laboratory evaluation will be needed if there is a change in manufacturer.

Fourth, we were unable to obtain one of the four control strains, *Escherichia coli* AR Bank #0348 as routine QC recommended by the CLSI due to restricted geographical access. The characteristic of this control strain demonstrates resistance to all three tested antimicrobial agents: CZA, ATM, and CZA–ATM. We were able to utilize our archived clinical isolates to replace this control strain.

With these in mind, future studies would benefit from including more varied MBL-producers to explore a wider application of these techniques. Previous scholars using the BMD reference method have found variable performance amongst *Enterobacterales* strains [18,23]. Since BDE is now an accessible standard of comparison method, non-reference laboratories have a lower barrier to entry for contributing to these evaluation studies.

## 4. Materials and Methods

### 4.1. Strain Collection

Forty archived *Enterobacterales* isolates that were non-susceptible to carbapenems were included in this study, including *Escherichia coli* (*n* = 35), *Enterobacter cloacae* complex (*n* = 2), *Klebsiella pneumoniae* complex (*n* = 2), and *Citrobacter freundii* complex (*n* = 1).

These isolates were collected in the period between 1 July 2022 and 25 October 2024 from three different tertiary regional hospitals located in New Territories in Hong Kong. They were isolated from various types of specimens, including urine (*n* = 17), a rectal swab (*n* = 11), ascitic fluid (*n* = 3), sputum (*n* = 3), a wound swab (*n* = 3), blood culture (*n* = 2), and tracheal aspirate (*n* = 1).

Identification of the isolates was performed by the Bruker (Billerica, MA, USA) MALDI-TOF Microflex system. The AST of Ertapenem and Meropenem was performed according to the CLSI M100 ED34:2024 [14]. The detection of carbapenemases was performed by either the NG-Test CARBA 5 (NG-Biotech, Guipry, France) lateral flow immunoassay or the Xpert Carba-R molecular assay (Cepheid, Sunnyvale, CA, USA). Thirty-nine isolates were NDM-E. One isolate was tested negative by CARBA-5 and did not harbor any known carbapenemases upon further multiplex PCR testing by the reference laboratory. The molecular targets of the multiplex PCR include Class A (KPC, SME, IMI, GES), Class B (IMP, VIM, SPM, NDM, SIM, GIM), and Class D (OXA-48) carbapenemases.

Experiments were performed in the Department of Microbiology, Prince of Wales Hospital. For patients with multiple isolates in the study period, only the first isolate from each episode of hospitalization was included.

### 4.2. The CLSI Aztreonam Plus Ceftazidime–Avibactam Broth Disk Elution Method (BDE)

The procedures were carried out according to Table 3D in the CLSI M100 guidelines [14]. In brief, 4 tubes of 5 mL cation-adjusted Mueller–Hinton broth (CAMHB) (Thermo Scientific, Waltham, MA, USA) were used for each of the isolates. Antibiotic disks (Thermo Scientific, U.S.) were added into 3 individual CAMHB tubes, one with a 30 μg ATM disk, one with a 30/20 μg ceftazidime–avibactam (CZA) disk, and one with a combination of both ATM and CZA disks. No antibiotic disk was added to the growth control. For each of the isolates, a standard inoculum was prepared by inoculating 3 to 5 bacterial colonies into 0.85% normal saline and adjusted to the 0.5 McFarland turbidity standard. Following this, 25 μL of the standardized inoculum was added into each of the 4 pre-labeled CAMHB tubes and incubated at 33–35 °C in ambient air for 16 to 20 h. The tubes were examined for turbidity after incubation by two independent scientists, and the concordance rate was recorded. All tests were run in parallel with quality control strains as recommended by the CLSI, except the *Escherichia coli* AR Bank #0348 strain that was replaced with an archived clinical isolate.

### 4.3. Disk Diffusion Method for Aztreonam–Avibactam

AZA DD was performed using Mueller–Hinton agar (MHA) and an antibiotic disk with a content of 30/20 µg AZA (Liofilchem, Roseto degli Abruzzi, Italy) and incubated at 35 °C for 18 to 20 h in ambient air. The zone diameter breakpoint was interpreted according to the EUCAST guidelines [16]. Disk diffusion zone sizes of ≥25 mm and <25 mm were interpreted as susceptible and resistant, respectively.

### 4.4. MIC Test Strip Method for Aztreonam–Avibactam

The AZA MTS method (0.016/4 to 256/4 μg/mL) (Liofilchem, Italy) was performed using MHA and incubated at 35 °C for 16 to 20 h in ambient air as per the manufacturer’s instructions. The MIC was interpreted according to the EUCAST guidelines [16]. MICs of ≤4 μg/mL and >4 μg/mL were interpreted as susceptible and resistant, respectively.

### 4.5. Gradient Strip Stacking Method

This was based on the method described by Davide et al. [11]. An ATM MTS (Liofilchem, Italy) was applied to MHA for 5 min first. The ATM MTS was then removed. A ceftazidime–avibactam (CZA) MTS (Liofilchem, Italy) was then applied onto the same location. The previous ATM MTS was placed on top of the CZA MIC test strip and incubated at 35 °C for 16 to 18 h in ambient air. The ATM MIC breakpoint in the presence of CZA MTS was interpreted as per the CLSI guidelines [14]. A MIC of ≤4 μg/mL and >4 μg/mL were interpreted as susceptible and resistant, respectively.

### 4.6. Statistical Analysis

Summary statistics were used for the description of background information.

Using BDE as the standard of comparison, the categorical agreement (CA), very major error (VME), major error (ME), and minor error rate of the isolates for the antibiotic combination of ATM and AVI were compared [24].

- CA refers to strains where the evaluated method yielded an AST result with the same categorical interpretation as the standard of comparison. CA ≥ 90% is considered acceptable.
- VME refers to strains tested resistant by the standard of comparison but tested susceptible by the method under evaluation. VME < 3% is considered acceptable.
- ME refers to strains tested susceptible by the standard of comparison but tested resistant by the method under evaluation. ME < 3% is considered acceptable.

The essential agreement (EA) between the MIC for AZA MTS and SS was calculated. Two tests were in EA for an isolate when the MIC obtained from both methods was within 1-fold dilution of each other. An EA rate of ≥90% was considered acceptable [17].

## 5. Conclusions

For NDM-producing *Enterobacterales*, AZA DD and the MIC test strips are viable CZA–ATM susceptibility testing options suitable for routine laboratory practices when BMD or BME are not accessible.

## Figures and Tables

**Table 1:** CA, VME, and ME using broth disk elution as the standard of comparison.

	AZA Disk Diffusion	AZA MIC Test Strip	Gradient Strip Stacking
CA	100%	100%	100%
VME	0%	0%	0%
ME	0%	0%	0%

Abbreviations; AZA: aztreonam–avibactam; CA: categorical agreement; VME: very major error; ME: major error; MIC: minimum inhibitory concentration.

**Table 2:** Comparison of aztreonam–avibactam MIC from MIC test strip and aztreonam MIC from gradient strip stacking test.

		ATM MIC (µg/mL)										
		0.016	0.032	0.064	0.125	0.25	0.5	1	2	4	8	16
Aztreonam–avibactam MIC (µg/mL)	0.032		1		1							
	0.064		4	6	5		3	1				
	0.125	1	1	2	3	2	1	1				
	0.25	1					1					
	0.5											
	1						3		1			
	2											
	4									1		
	8											
	16											
	32											
	64											
	128											1

## Data Availability

The original contributions presented in this study are included in the article. Further inquiries can be directed to the corresponding author.
